# Platelet-Rich Plasma in Equine Osteoarthritis: A Systematic Review of Clinical and Experimental Evidence

**DOI:** 10.3390/ani15182647

**Published:** 2025-09-09

**Authors:** Jorge U. Carmona, Catalina López

**Affiliations:** 1 Grupo de Investigación Terapia Regenerativa, Departamento de Salud Animal, Universidad de Caldas, Calle 65 No 26-10, Manizales 170004, Colombia; 2 Grupo de Investigación Patología Clínica Veterinaria, Departamento de Salud Animal, Universidad de Caldas, Calle 65 No 26-10, Manizales 170004, Colombia; catalina.lopez@ucaldas.edu.co

**Keywords:** equine osteoarthritis, orthobiologics, platelet-rich plasma, intra-articular treatments, regenerative medicine

## Abstract

Osteoarthritis (OA) is a major cause of equine lameness, with few effective disease-modifying treatments. This systematic review, conducted following the Preferred Reporting Items for Systematic Reviews and Meta-Analyses (PRISMA) guidelines, evaluated the efficacy of platelet-rich plasma (PRP) for equine OA by analyzing 11 studies (6 clinical, 5 experimental) identified through Web of Science, Scopus, and PubMed (2000–2024). The screening process identified 252 records, of which 136 were duplicates and 105 were excluded based on predefined criteria. The analysis showed that intra-articular PRP injections are generally safe, with transient synovial inflammation occurring mainly when PRP was activated with bovine thrombin. Both leukocyte-rich (L-PRP) and leukocyte-poor (P-PRP) formulations exhibited comparable efficacy, though optimal platelet concentrations (423–658 × 10^3^/μL) and dosing regimens remain undefined. A PRISMA-based quality assessment highlighted substantial variability in study design, with clinical trials constrained by small sample sizes and high risk of bias. Experimental studies confirmed PRP’s biological activity but showed inconsistencies in preparation methods. The findings indicate that PRP activation is unnecessary and may even be pro-inflammatory, that multiple injections could improve outcomes, and that reporting of cellular composition is inconsistent across studies. The PRISMA framework identified critical evidence gaps, particularly regarding long-term efficacy and protocol standardization. These results emphasize the need for PRISMA-compliant randomized controlled trials featuring standardized PRP protocols, validated outcome measures, and extended follow-up periods to establish evidence-based guidelines for managing equine OA.

## Simple Summary

Osteoarthritis (OA) is the leading cause of lameness in horses, often resulting in loss of athletic performance, early retirement, or euthanasia. Current treatments may ease symptoms but rarely stop the disease from worsening. Platelet-rich plasma (PRP) is a blood-derived product containing platelets and natural healing factors that may reduce joint inflammation and support tissue repair. In this systematic review, we analyzed all available clinical and experimental studies on PRP for equine OA. Eleven studies met the inclusion criteria. The results show that intra-articular PRP injections are generally safe, with only mild and short-lasting inflammation reported in some cases, usually when PRP was activated with bovine thrombin. Both main types of PRP—those with and without white blood cells—appeared to provide similar improvements, such as reduced lameness and better joint function. However, the optimal platelet concentration, need for activation before injection, and best dosing schedules remain undefined. Most benefits were observed within the first months, but long-term results were less frequently reported. Our findings highlight important evidence gaps and call for future well-designed clinical trials with standardized PRP preparation, clear reporting of its composition, and extended follow-up to develop reliable, evidence-based guidelines for veterinarians and horse owners.

## 1. Introduction

Osteoarthritis (OA) is the most common cause of lameness in horses worldwide [1,2,3]. Each year, hundreds of millions of dollars are spent on managing the symptoms of this chronic musculoskeletal condition [4]. To date, no highly effective therapy has been identified that can halt the progression of OA to an end-stage degenerative condition [5,6]. Supportive measures such as corrective farriery, conventional therapies, controlled exercise, and training may slow disease progression in some cases, but they do not provide a cure.

However, the results of these approaches are variable, and their effectiveness typically declines over time. Consequently, affected horses are often withdrawn from athletic activities, and in some cases, euthanasia becomes necessary [7,8]. In addition, long-term use of NSAIDs and corticosteroids has been associated with gastrointestinal, renal, and cardiovascular side effects that may compromise patient health [9].

Over the past two decades, regenerative medicine has advanced in both human and veterinary fields [10,11,12,13,14]. In horses, various approaches have been employed, including mesenchymal stem cells, purified recombinant growth factors (GFs), and platelet-rich hemocomponents such as platelet-rich plasma (PRP), platelet lysate, and lyophilized platelets [15,16,17,18]. Stanozolol has also been used in equine arthropathies. Although not a true regenerative therapy, it is widely applied in practice for its potential to modulate joint metabolism, though its efficacy and safety remain debated [19,20].

PRP is a suspension of platelets and leukocytes in plasma, with concentrations that vary depending on the preparation method [14,21]. It can be classified as either pure PRP (P-PRP) or leukocyte-rich PRP (L-PRP) [22,23]. In horses, P-PRP is characterized by low-to-moderate platelet concentrations—equal to or slightly higher than those in whole blood—and minimal leukocyte content. In contrast, L-PRP contains higher platelet counts along with measurable leukocyte concentrations [14,22,23]. Following injection, PRP polymerizes into a platelet-rich gel (PRG), which triggers the release of growth factors and anti-inflammatory cytokines. This cascade promotes recruitment of local stem cells and supports tissue regeneration and repair [22,23].

A recent systematic review and meta-analysis [24] on the use of PRP for treating both OA and septic arthritis (SA) in horses covered a wide variety of PRP-related products, such as platelet lysates, and freeze-dried platelets and focused on clinical outcomes despite variations in study methods. In contrast, the present review takes a more focused approach by examining only PRP’s role in equine OA to better understand its specific mechanisms and clinical benefits for OA.

Peng et al. [24] emphasized PRP’s wide-ranging therapeutic potential, including its antimicrobial effects on SA. However, our review prioritized standardization and critically evaluated inconsistencies in how PRP was prepared, such as differences in centrifugation protocols and activation methods. It also addresses key gaps in OA treatment, like identifying the optimal platelet concentration and assessing long-term effectiveness. Unlike Peng et al. [24], this review narrowed its scope to PRP and closely related products such as APS, excluding other platelet-derived preparations (e.g., platelet lysates or lyophilized platelets). This focus allows for more precise conclusions regarding the role of intra-articular PRP in equine OA, while acknowledging that APS has distinct biological features that must be interpreted with caution.

The aim of this study was to conduct a systematic review of clinical and experimental data on PRP for equine OA, with particular attention paid to standardization and reproducibility. Specifically, we sought to answer the following questions: (1) What adverse effects have been reported with intra-articular injections (IAIs) of PRP in horses, and what safety profile can be established from available studies? (2) What types of PRP (P-PRP vs. L-PRP) are most commonly used and effective? (3) What is the optimal concentration of platelets and leukocytes in PRP for treating equine OA? (4) Should PRP be activated before IAI, and if so, what is the most effective method? (5) What is the ideal dosing regimen (volume, frequency) for PRP in equine OA? (6) What are the short- (1–3 months), medium- (3.1–12 months), and long-term (≥12.1 months) clinical outcomes of PRP treatment? By addressing these questions, this review aims to inform both future research and clinical practice, moving toward standardized, evidence-based PRP protocols.

## 2. Materials and Methods

This systematic review did not involve animal experimentation or clinical procedures and therefore did not require ethics committee approval.

### 2.1. Study Design

This review followed the Preferred Reporting Items for Systematic Review and Meta-Analyses (PRISMA) 2020 guidelines [25,26] and employed the Population, Intervention, Comparison, Outcome (PICO) framework [27,28,29].

### 2.2. Search Strategy

Web of Science, Scopus, and PubMed Databases were used for this review. The study timeframe was set from January 2000 to December 2024. The search terms used were: (“platelet-rich plasma” OR “platelet concentrate*” OR “autologous protein solution”) AND (horse* OR equine OR equus OR equid*) AND (osteoarthritis OR “degenerative joint disease*” OR “joint disease*” OR arthritis OR cartilage* OR chondro* OR “joint degeneration”).

### 2.3. PICO Framework

Table 1 summarizes the PICO framework applied to each of the six research questions. Of note, this systematic review focused only on papers related to the use of PRP as a clinical treatment for horses with naturally occurring OA, as an experimental treatment for induced OA, or as intra-articular injection (IAI) in normal equine joints.

### 2.4. Study Selection

Databases were independently accessed by C.L. and J.U.C., and search results were uploaded to EndNote Web (Clarivate, London, UK) for duplicate removal.

The obtained registers were evaluated independently by each author, considering the title, keywords, and abstract information. Each register was assessed based on both inclusion and exclusion criteria. For this review, only articles that met the following inclusion criteria were included: (1) Studies focusing on PRP in horses, specifically randomized controlled trials (RCTs), non-randomized controlled trials (No-RCTs), case series (CS), and controlled experimental studies (CESs) published in peer-reviewed scientific journals in English. (2) Horses treated with any type of PRP preparation, including pure P-PRP or L-PRP. (3) Studies involving horses with naturally occurring OA or experimental horses with OA, and (4) availability of the full text of the manuscript.

Inclusion criteria also considered the following specific aspects, summarized in Table 2.

The exclusion criteria were: (1) Narrative or systematic reviews, meta-analyses, commentaries, letters to the editor, abstracts, and case reports. (2) Studies conducted in other species. (3) Studies that used PRP as OA treatment in combination with other products and (4) studies focused on other pathologies in organs other than joints.

Titles, abstracts, and keywords were screened independently by both authors based on predefined inclusion and exclusion criteria. Subsequently, both researchers presented their results regarding the screened documents in a meeting. Any disagreements about document selection were resolved by consensus between the authors.

### 2.5. Data Extraction

After analysis, the selected articles were summarized in three tables, one describing the CSs, another showing the RCTs in which this hemocomponent was used as a treatment for naturally occurring OA, and the other describing the CESs in healthy horses treated with IAIs of PRP. The tables included the following information: authors and date of publication, study design and type of PRP evaluated, results, observations of the studies, and overall outcome.

Outcomes were assessed in the short term (1–3 months), medium term (3.1–12 months), and long term (≥12 months), based on the horses’ ability to return to sport or work, as well as relevant paraclinical data (e.g., radiological findings, synovial or plasma biomarkers). Overall outcomes were categorized as positive (lameness reduction, return to work, or improved synovial fluid parameters), negative (worsened lameness or joint effusion), or neutral (no significant changes).

### 2.6. Quality Assessment of Platelet-Rich Plasma

Selected documents were analyzed according to a slightly modified set of criteria originally outlined in the Platelets editorial policy for PRP studies [30]. This policy, itself derived from the broader guidance of the Platelet Physiology Subcommittee of the Scientific and Standardization Committee (SSC) of the International Society on Thrombosis and Haemostasis (ISTH) [31], established the minimum methodological items that PRP reports should include to ensure transparency and reproducibility.

For the purposes of this review, we adapted these requirements into 10 characteristics (C1–C10): C1. Source of blood (autologous [AUT] or allogeneic [ALL]). C2. Anticoagulant, volume, and age of blood used for PRP preparation. C3. Method used to prepare PRP. C4. Centrifugation conditions (g-force, temperature, and duration) used in laboratory or commercial devices. C5. Detailed description of PRP harvesting (e.g., buffy coat or PRP supernatant), including commercial device brand if applicable. C6. Cellular composition of whole blood (platelet, white blood cell, and red blood cell counts). C7. Quality assessment of PRP (cell content, platelet activation status, platelet-specific proteins, and growth factor content). C8. Platelet concentration factor and yield. C9. PRP activation prior to use, including substance used. C10. Method and number of in vivo applications, specific sites of administration, and volume of PRP delivered.

It should be noted that the original Platelets editorial policy listed 11 criteria. In our adaptation, the apparent reduction to 10 was the result of restructuring rather than exclusion of methodological domains. Specifically, the original items 5 (commercial device details) and 6 (harvesting method) were merged into a single criterion (C5), since both referred to how PRP was collected and whether a commercial kit was used. Conversely, the original item 7 (cellular content of whole blood and PRP) was divided into two criteria in our framework (C6 and C8), to separately capture baseline blood counts and final PRP yield, which are often inconsistently reported. Finally, aspects related to growth factor quantification—although seldom available in equine studies—were integrated into C7 rather than treated as an independent item, to avoid systematically penalizing most reports.

Thus, the adjustment from 11 to 10 reflects restructuring for clarity and reproducibility, while preserving the essential methodological dimensions of the original checklist. Each characteristic was scored from 0 to 10, resulting in a total score between 0 and 100. Studies with a score < 40 were classified as having a poor methodological description, those with a score between 41 and 80 as moderate, and those with a score > 81 as good. This semiquantitative framework was first applied in a previous systematic review of PRP for equine tendon and ligament injuries [32], and here it was adapted to the context of equine osteoarthritis.

### 2.7. Bias Risk Assessment

The risk of bias in included studies was independently assessed by J.U.C. and C.L., following PRISMA guidelines [25,26]. CSs were evaluated according to the ROBINS-I framework for non-randomized studies [33], covering seven domains: (1) confounding bias, (2) selection bias, (3) bias in intervention classification, (4) deviations from intended interventions, (5) missing data, (6) outcome measurement, and (7) selective reporting. Each study was rated as low, moderate, serious, or critical risk of bias in each domain [33].

Randomized clinical trials and experimental controls were assessed using the RoB2.0 tool [34], covering five domains: (1) bias from the randomization process, (2) deviations from intended interventions, (3) missing outcome data, (4) outcome measurement, and (5) selective reporting. Each study was rated as low risk, some concerns, or high risk for each domain [34].

To ensure objectivity, discrepancies between assessors were resolved through discussion until consensus was reached, ensuring uniform application of bias assessment criteria. Methodological limitations identified included non-randomized study designs, lack of evaluator blinding, and incomplete reporting of treatment results. The review also acknowledged inherent constraints, including variability in PRP processing and heterogeneous outcome measures. These differences were carefully considered in data interpretation. Bias analysis results were visually presented using the ROVIS platform, incorporating ROBINS-I and RoB2.0 frameworks (https://mcguinlu.shinyapps.io/robvis/, accessed on 7 July 2025) [35].

## 3. Results

### 3.1. Study Selection

**Figure 1:**
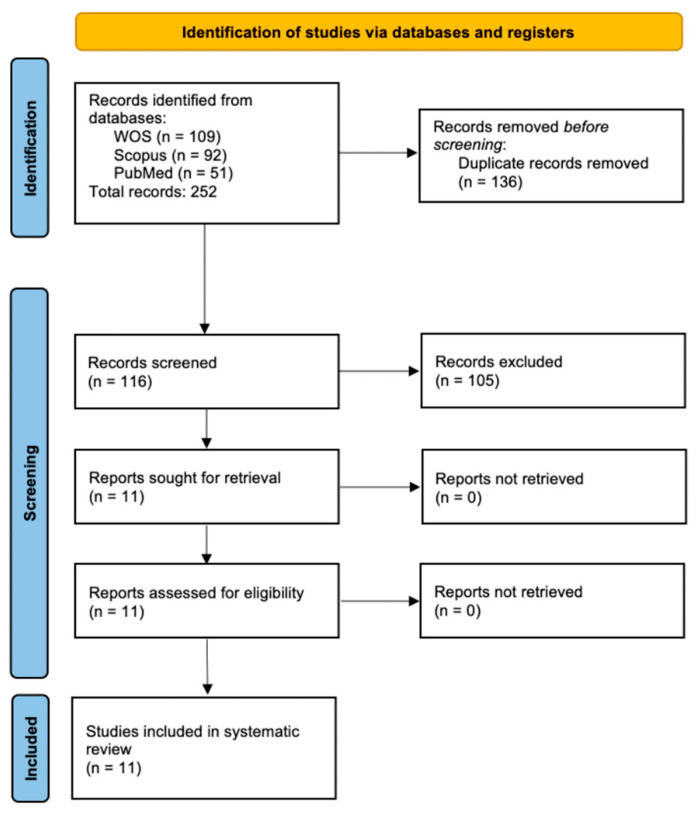
Flow chart of the study according to PRISMSA criteria.

During the search for scientific articles, initially 252 documents were found (WOS, 109 records; SCOPUS, 92 records; and PubMed, 51 records), of which 136 articles were duplicated for an initial screening of 116 registers. Subsequently, through the analysis of the title, abstract, and keywords, 105 articles were excluded, for a total of 11 scientific articles that met the inclusion criteria [36,37,38,39,40,41,42,43] (Figure 1).

### 3.2. Population and Intervention Characteristics

Of the 11 selected documents, 4 were CSs [36,37,38,40] (Table 3), 2 were RCTs [39,44] (Table 4) and 5 were CESs [41,42,43,45,46] (Table 5). In the CSs and RCTs, a total of 64 horses with OA received IAIs of various types of PRP, with 25 horses acting as controls (20 with OA and 5 clinically healthy). Additionally, the CESs included 38 horses, of which 20 were clinically healthy at the time of PRP IAI [41,42,43,45], while 18 horses were evaluated after induced tarsocrural synovitis via cytokine stimulation [46].

#### 3.2.1. Case Series and RCTs

Forty-nine horses were treated with several PRP products in the 4 CSs [36,37,38,40], while 35 horses were included in the treatment group of the RCTs [39,44]. In these studies, the joints treated were fetlocks (both metatarso- and metacarpo-phalangeal joints), carpus, tarsus, and stifles, all considered high motion joints [44]. The two RCTs in this review included a trial with an OA group treated with PRP (n = 5), and control healthy group treated with saline (n = 5) [39], while a RCT included an experimental group of 20 horses with naturally occurring OA of several high motion joints and a control group of 20 horses with similar joint conditions treated with IAI of saline solution [44].

#### 3.2.2. Controlled Experimental Studies

Thirty-three horses were used in the 5 CESs included in this review [41,42,43,45,46]. Three out of 5 studies were mainly focused in evaluating the local effects of the IAI of PRP in normal fetlock joints [41,42,43]. However, two works were performed using the same animals and synovial fluid samples [41,42]. Additionally, one study [46] evaluated the effect of PRP in an equine model of tarsocrural synovitis induced with recombinant equine IL-1β, while another study evaluated the metabolic effect of PRP compared to saline solution and triamcinolone acetonide without considering clinical or biomarker indicators of joint inflammation [45].

### 3.3. Outcomes

#### 3.3.1. Safety (Objective 1)

Three CESs [41,42,43] and one clinical study [39] investigated joint reactions to PRP injections by examining clinical parameters and changes in synovial and plasma biomarkers, including growth factors, cytokines, and serum amyloid A (SAA). Mirza et al. [38] evaluated the effect of a single PRP injection on biomechanical parameters related to joint inflammation, while one experimental study assessed the local and systemic effects of PRP compared to saline in a cytokine-induced synovitis model [46]. PRP induced transient synovial fluid inflammatory changes in normal equine joints [41,42,43], which were more pronounced when PRP was activated with bovine thrombin [41,42].

#### 3.3.2. Platelet-Rich Plasma Types (Objective 2)

According to Dohan Ehrenfest et al.’s classification system for platelet concentrates [22,23], the types of PRP evaluated in the 11 studies included three L-PRP products and two P-PRP types. Among the L-PRP studies, four obtained the platelet concentrate using a gravitational filtration method [38,39,41,42], and two used a double-tube centrifugation method [45,46]. In contrast, P-PRP was obtained through double centrifugation in one study [47] and with a semi-automatic kit in another study [40].

#### 3.3.3. Platelet-Rich Plasma Composition (Objective 3)

The platelet and leukocyte concentration of the different PRPs used was described in all studies. The platelet concentration ranged from 423–658 × 10^3^ cytoplasmic fragments/mL for the two types of PRP [37,38,39,40,41,42,43]. The concentration of leukocytes was not detected or was very low in the P-PRPs [37,40], while in L-PRPs it ranged from 8.36–75 × 10^3^ cells/μL [36,38,39,41,42,43,44]. As for the concentration of growth factors in PRP, this was described in 2 experimental studies [41,42] and in two clinical studies [36,37], with concentrations of TGF-β_1_ between 954 and 3830 pg/mL and PDGF-BB between 450 and 3811 pg/mL [36,37,41,42].

#### 3.3.4. Platelet-Rich Plasma Activation (Objective 4)

PRP activation was performed in only three studies: two experimental [41,42] and one clinical [36]. Experimental studies used calcium chloride and bovine thrombin as activators [41,42], while the clinical study used only calcium chloride [36].

#### 3.3.5. Platelet-Rich Plasma Dosing (Objective 5)

PRP volumes in experimental studies ranged from 2.5 to 4 mL per injection [42,43,44], while in CSs and RCTs, administered volumes ranged from 3 to 25 mL [41,42,43], while in CSs and RCTs, administered volumes ranged from 3 to 25 mL [36,37,38,39,40]. Eight studies used a single IAI of PRP, (five CEs [41,42,43,45,46] and three clinical [38,39,44]). In contrast, two clinical studies administered three consecutive injections at 2-week intervals [36,37], and one study administered two injections at the same interval [40].

#### 3.3.6. Platelet-Rich Plasma Efficacy (Objective 6)

Outcome evaluation in CSs and RCTs ranged from 2 [40] to 12 months [37,44], with one study reporting only a 5-day post-treatment outcome [39]. The overall clinical outcome was generally positive (Table 3 and Table 4), though one CS reported 80% success at one year [37], and one RCT reported only 20% success (6/20 horses) over the same period. Notably, one case series observed a recurrence of clinical signs in OA-affected horses eight months after PRP treatment, suggesting that repeated administrations may be necessary to sustain therapeutic benefits and maintain symptom-free periods [36].

On the other hand, the outcome time in the experimental studies ranged between 4 [41,42] and 28 days [43]. Those studies that mainly evaluated the local reaction of joints to PRP administration were classified with a neutral overall outcome [41,42,43,46], while the general outcome of one experimental study that evaluated the systemic metabolic effects of the intraarticular injection of PRP in comparison to corticosteroid was scored as positive [45].

### 3.4. Quality Assessment of Platelet-Rich Plasma

A summary describing the quality process for PRP procurement according to the ISTH criteria [31] is presented in Table 6 and Table 7. The semiquantitative analysis used to classify the methodological description of PRP production and quality in the studies showed that the scores ranged from 35 [45] to 100 [44] with a median score of 80. One out of 11 studies presented a poor methodological description of PRP production and quality [45]. Five out of 11 studies showed a moderate methodological description of PRP [36,37,41,43,46], while 5 of 11 studies had a good methodological description of PRP of production and quality [38,39,40,42,44]. The lowest-scored characteristics across studies were C6 (measurement of cellular content in whole blood, including platelets, WBCs, and RBCs) and C8 (platelet concentration factor and yield) (Table 8).

### 3.5. Bias Risk Assessment

#### 3.5.1. Case Series and RCTs

**Figure 2:**
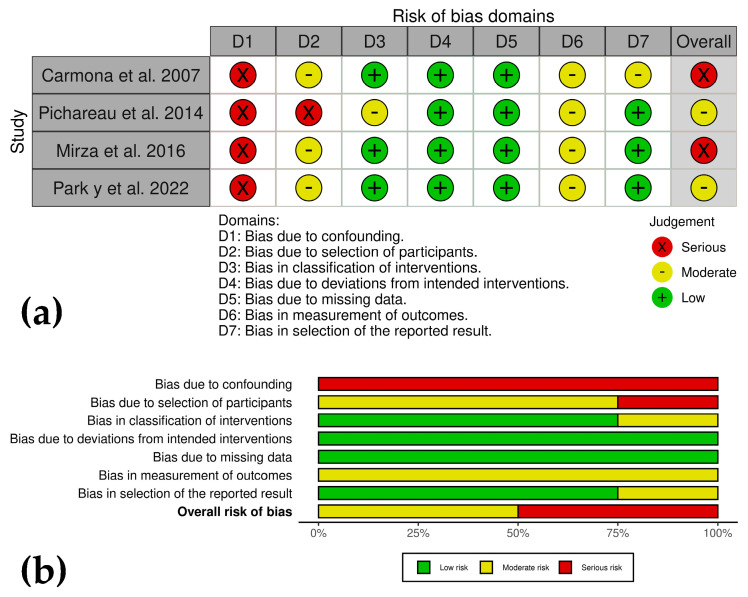
(**a**) Traffic light plots showing domain-level judgments for each outcome in the case series. (**b**) Weighted bar plots of the distribution of risk of bias ratings within each domain.

The CSs analyzed using the risk of bias in non-randomized studies of interventions (ROBINS-I) tool demonstrate varying levels of bias, with common limitations including small sample sizes, lack of randomization and blinding, and reliance on subjective outcomes [36,37,38,40]. While some studies show moderate risk due to objective measures supporting findings, others exhibit serious risks due to confounding and selection biases. Across all studies, the absence of control groups and unblinded assessments reduces reliability (Figure 2a,b). For instance, Mirza et al. [38] and Park et al. [40] highlight the importance of objective measures but call for further controlled studies. Similarly, Pichereau et al. [37] and Carmona et al. [36] underscore the preliminary nature of their findings and recommend larger, more rigorous trials.

**Figure 3:**
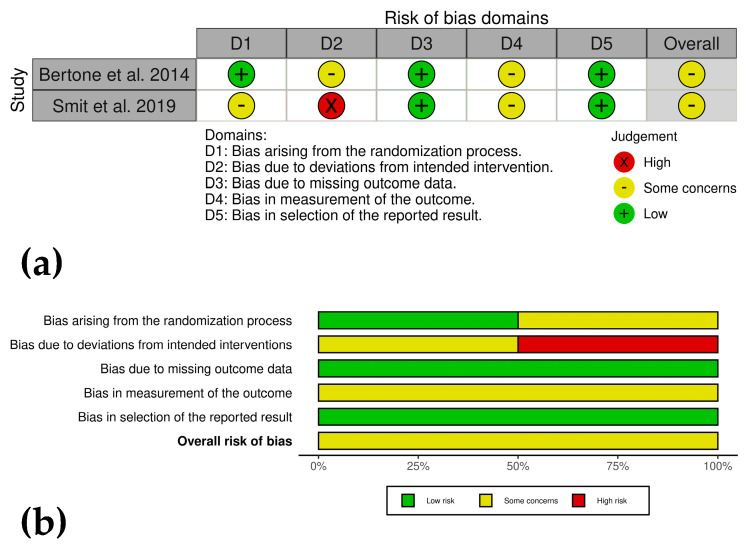
(**a**) Traffic light plots showing domain-level judgments for each outcome in the clinical studies. (**b**) Weighted bar plots of the distribution of risk of bias ratings within each domain.

The clinical studies (analyzed using the RoB2.0 framework) by Bertone et al. [44] and Smit et al. [39] exhibited some concerns regarding risk of bias, primarily due to lack of blinding, and subjective outcome measures. Both studies demonstrated strengths in complete data reporting and objective outcome measures. However, the absence of detailed randomization and blinding raised concerns about potential bias (Figure 3a,b).

#### 3.5.2. Controlled Experimental Studies

**Figure 4:**
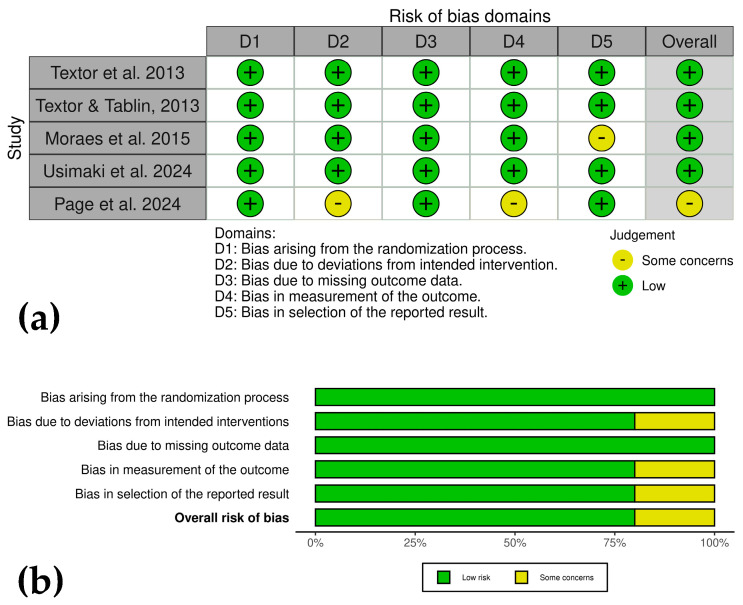
(**a**) Traffic light plots showing domain-level judgments for each outcome in the experimental studies. (**b**) Weighted bar plots of the distribution of risk of bias ratings within each domain.

The analysis showed variable risk of bias across studies. Textor et al. [41], Textor and Tablin [42], and Usimaki et al. [46] demonstrated low risk in all domains. Moraes et al. [43] showed low risk but raised concerns about reported result selection, while Page et al. [45] exhibited some concerns due to deviations from interventions and outcome measurement. Strengths included robust randomization, minimal missing data, and objective measures like force plate analysis (Figure 4a,b).

## 4. Discussion

The primary objective of this systematic review was to evaluate the efficacy and safety of the IAI of PRP in horses with OA. By synthesizing evidence from eleven included studies, we aimed to address six key research questions based on the PICO framework. The findings indicated that PRP improved clinical parameters such as lameness scores and joint effusion in both naturally occurring OA and experimental models. However, considerable heterogeneity in PRP preparation methods, activation protocols, and administration strategies limits the ability to draw definitive conclusions. Below, we discuss the results in detail, focusing on the six research questions previously outlined.

### 4.1. Safety of PRP in Equine OA (Objective 1)

The reviewed studies consistently demonstrated that intra-articular PRP injections are safe for both healthy horses and those with OA when prepared and administered under aseptic conditions [47]. Both L-PRP and P-PRP formulations—whether obtained by gravity filtration [38,39,41,42], semi-automated kits [40,44,45,46], or double-tube centrifugation [36,37,43]—showed excellent safety profiles. Transient synovial inflammation was primarily observed when PRP was activated with bovine thrombin, as evidenced by increased leukocyte counts and inflammatory cytokines (e.g., TNF-α and IL-6) [41,42,43]; however, no long-term adverse effects or systemic complications were reported. These findings are supported by comprehensive evaluations from two experimental studies [41,42] in healthy joints and Smit et al. [39] in OA-affected horses, which included analysis of synovial fluid cytology, growth factor concentrations, pro-inflammatory cytokines, and acute phase proteins like SAA. The collective data confirm PRP’s safety while demonstrating its measurable biological effects at the joint level [39,41,42,48].

### 4.2. Efficacy of PRP Types: L-PRP vs. P-PRP (Objective 2)

Most CESs in healthy horses [38,41,42] and in one clinical trial in OA-affected horses [39] utilized L-PRP preparations obtained through gravitational filtration [14,22,23]. These L-PRP formulations exhibited high platelet and growth factor concentrations while maintaining therapeutic efficacy. Furthermore, a clinical study assessing L-PRP produced by a semiautomated kit reported elevated concentrations of anti-inflammatory cytokines, suggesting additional benefits beyond standard preparations [44]. Parallel clinical evidence shows that P-PRP preparations, typically derived through double centrifugation methods, have also been successfully employed in equine OA treatment [37,40]. Importantly, both L-PRP and P-PRP formulations showed comparable clinical benefits including lameness reduction and improved joint function across studies.

This collective evidence suggests that while preparation methods differ significantly between formulations, the choice between them should be guided by specific clinical contexts rather than the presumed superiority of one type over another. The comparable outcomes observed with both formulations suggest that factors beyond leukocyte content—such as platelet concentration, growth factor profile, and administration protocol—may be equally important determinants of therapeutic success.

Although APS was included in our search strategy and therefore appears in this review, it should be noted that APS is not strictly equivalent to conventional PRP. APS is obtained through a two-step processing method, starting from L-PRP and subsequently filtering and concentrating soluble anti-inflammatory proteins such as IL-1Ra, sTNF-R, and IL-10 [16,49], while significantly reducing the number of platelets and leukocytes injected. Thus, its biological mechanism differs from that of PRP, where the cellular fraction (containing platelets and leukocytes) and their growth factor release play a central role. Nevertheless, APS was retained in this review because, up to the point of filtration, it remains a living biomaterial derived directly from whole blood, which sets it apart from other platelet-derived products such as platelet lysates or lyophilized platelets, in which cell viability is completely lost. For this reason, the interpretation of APS results should be made with caution, and comparisons with PRP studies should acknowledge this distinction. Future systematic reviews specifically addressing APS and other orthobiologics are warranted to clarify their relative clinical utility in equine OA.

### 4.3. Optimal Platelet and Leukocyte Concentrations (Objective 3)

Platelet concentrations in the evaluated PRP preparations ranged from 423–658 × 10^3^/μL, with leukocyte counts higher in L-PRP (8.68–75 × 10^3^/μL) than in P-PRP. Growth factors, including TGF-β_1_ and PDGF-BB, and anti-inflammatory cytokines were detected at bioactive concentrations, supporting PRP’s regenerative potential [36,37,38,39,40,41,42,43,44]. However, the lack of dose–response studies precludes definitive conclusions about optimal concentrations. Future research should standardize reporting to correlate cellular composition with clinical outcomes.

Excessive platelet counts (>1000 × 10^3^/µL), especially with leukocytosis, may promote inflammation, while moderate enrichment (2–5-fold) seems more favorable for repair. The lack of standardized reporting on leukocyte counts limits cross-study comparability, but current evidence suggests that L-PRP may elicit stronger early inflammatory responses compared to P-PRP, without consistent superiority in long-term outcomes [50].

Taken together, these findings suggest that platelet and leukocyte counts may influence early inflammatory responses, but current data are insufficient to define thresholds for optimal clinical outcomes.

### 4.4. PRP Activation: Necessity and Methods (Objective 4)

Activation with bovine thrombin exacerbated synovial inflammation, whereas calcium chloride or non-activated PRP elicited milder responses [41,42]. Clinical studies using non-activated PRP reported positive outcomes, suggesting that pre-injection activation is unnecessary and may even be detrimental. These findings could challenge the routine use of exogenous activators in equine OA treatment.

Activation methods directly influence the kinetics of growth factor release [48,51]. Calcium chloride induces rapid degranulation, while bovine thrombin can additionally trigger pro-inflammatory cytokine release, explaining the transient synovitis reported in some equine studies [42]. Non-activated PRP relies on endogenous activation within the joint or tendon microenvironment, potentially offering a more physiological release profile with fewer adverse events.

### 4.5. Dosing Regimens: Volume and Frequency (Objective 5)

The systematic review revealed substantial variability in PRP dosing protocols for equine OA treatment, with limited guidance regarding optimal injection volumes and administration frequency. Injection volumes varied significantly by joint, ranging from 2.5 to 10 mL for fetlocks [36,37,39,40,41,42,43], 10 mL for carpal joints [38], and up to 25 mL for femorotibial articulations [36]. Importantly, these volume ranges appeared well-tolerated, with no complications directly attributed to injection volume and post-administration synovial effusion appeared to be primarily associated with PRP activation methods rather than injection volume [41,42].

Treatment frequency also varied across studies. Some protocols achieved clinical improvements with a single injection [38,39,44], whereas others employed multiple administrations, either two injections [40] or three treatments at 15-day intervals [36,37]. Multi-injection protocols often demonstrated greater efficacy, showing significant reductions in lameness in short-[40], mid-[36], and long-term follow-ups [37]. However, single injections using a L-PRP approach also produced modest long-term benefits [44].

This variability in dosing (volume and frequency) highlights key gaps in defining optimal PRP protocols. While current evidence confirms that various dosing strategies can be effective, it highlights the urgent need for standardized volume recommendations based on joint characteristics, accompanied by well-controlled comparative studies to establish evidence-based guidelines. Such research should specifically examine the relationship between injection parameters and clinical outcomes while considering practical factors such as treatment cost and patient compliance.

### 4.6. Clinical Outcomes (Objective 6)

Short-term outcomes (1–3 months) demonstrated significant improvements, including reduced lameness and synovial effusion [36,40]. By 12 months, 20% [44] to 80% [37] of treated horses had returned to work. However, long-term follow-up data (≥12.1 months) remained scarce, and few studies evaluated radiographic OA progression. Additionally, the lack of standardized outcome measures and control groups in many trials complicates the assessment of PRP’s disease-modifying effects.

While the answers to the six proposed questions are based on the available evidence, we acknowledge that the limited data may not provide definitive conclusions. To enhance clarity and persuasiveness, we have reorganized the responses to align directly with the PICO framework, ensuring systematic referencing of the included studies.

### 4.7. Platelet-Rich Plasma Quality Assessment

The analysis of PRP studies reveals significant standardization challenges, with methodological quality scores ranging from 35 to 100 (median: 80) (Table 7). While some studies, like Bertone et al. [44], met all criteria, most neglected critical parameters; particularly, baseline cellular composition (C6) and platelet yield efficiency (C8), with 6/11 and 5/11 studies scoring zero, respectively. This pattern aligns with well-documented limitations in human PRP research [52,53], where insufficient cellular and molecular profiling hinders evidence synthesis. Even high-scoring studies (≥80) often prioritized procedural basics (e.g., PRP type) over comprehensive characterization, undermining comparability. RCTs scored higher (90–100) than case series (35–90), but gaps persisted across all study types, reflecting systemic reporting deficiencies rather than isolated flaws.

These findings highlight the urgent need for standardized guidelines, especially for cellular and yield metrics (C6/C8), to address barriers in clinical translation. Future research must prioritize these parameters, with consensus efforts involving multidisciplinary stakeholders to ensure rigorous yet practical implementation. Such standardization would enhance therapeutic evaluation and cross-study comparisons, advancing PRP’s clinical potential while overcoming existing limitations in evidence synthesis [52,53].

### 4.8. Limitations of the Systematic Review

This systematic review highlights several important limitations that should be acknowledged. The limited number of included studies (particularly the absence of RCTs) underscores a significant gap in the literature and the need for further high-quality research on PRP treatment for equine OA. While we expanded our search timeframe to December 2024 and included ‘autologous protein solution (APS)’ in the search algorithm to identify three additional studies (totaling 11 evaluated), this review still faces important limitations.

Performing a quantitative synthesis or meta-analysis was not possible due to the high heterogeneity between study designs, outcome measures and PRP preparation methods [52,53,54]. The lack of standardized reporting, particularly regarding platelet concentrations, growth factor concentrations, and clinical scoring systems, prevented the statistical pooling of data and limited the ability to draw definitive conclusions about PRP’s efficacy. Another critical limitation is the small sample sizes in the included studies, with group sizes typically ranging from 4 to 20 horses. These small cohorts limit statistical power and external validity [32,55,56].

The high heterogeneity across studies further complicates interpretation. Variability in PRP preparation methods (i.e., centrifugation protocols, commercial kits, activation techniques) resulted in inconsistent platelet and leukocyte concentrations, as well as growth factor and anti-inflammatory cytokine profiles. Dosing regimens also varied widely, with injection volumes ranging from 2.5 to 25 mL and treatment frequencies ranging from single to multiple doses.

Evaluating the reporting quality of PRP studies remains a challenge. The scoring system applied in this review was adapted from the minimum reporting criteria proposed in the Platelets editorial policy [30], itself based on ISTH SSC guidance [31], and had been successfully implemented in a prior equine-focused systematic review [32]. This semiquantitative framework offers a reproducible way to assess methodological transparency and facilitates comparison across heterogeneous studies, while also highlighting areas where standardization is urgently needed. At the same time, we recognize its limitations: the equal weighting of criteria, the use of exploratory thresholds, and the inherent subjectivity of scoring. For these reasons, the system should be interpreted with caution. Nevertheless, it proved useful in identifying major gaps—such as inconsistent data on cellular composition and activation protocols—and in guiding research priorities. We view this approach as a first step, and further refinement, ideally in collaboration with international peers working on PRP in equine and other species, will be necessary to validate and strengthen its robustness.

Long-term data were scarce, with only two studies reporting outcomes beyond 12 months [37,44]. This gap leaves PRP’s durability and potential disease-modifying effects unclear, as most studies focused on short-term synovial fluid changes or lameness reduction rather than structural joint preservation. The risk of bias in clinical studies was another concern. Non-randomized designs, particularly case series, dominated the literature, introducing selection bias. Many studies also lacked blinding, with subjective outcomes assessed by unblinded clinicians or owners, increasing the risk of performance and detection bias. Few studies included placebo or active comparators, weakening causal inferences.

Inconsistent reporting of PRP composition further hindered reproducibility. Critical parameters such as platelet yield efficiency and baseline whole-blood cellular counts were frequently omitted, and only a minority of studies provided growth factor data. Potential publication bias may also have skewed results, as negative or neutral findings are less likely to be published. Additional limitations include a lack of focus on specific joints or OA stages. Most studies treated high-motion joints like fetlocks, with limited data on other articulations or variations in OA severity. Finally, no study evaluated the cost-effectiveness of PRP compared to conventional therapies, which is an important consideration for clinical adoption.

Despite efforts to broaden the search strategy, this systematic review remains constrained by the small number of studies, methodological heterogeneity, and reliance on observational data, which may affect the generalizability of the findings. Future research should prioritize standardized PRP protocols, larger RCTs (particularly double-blind designs), long-term follow-up, and economic analyses. Only through such rigorous studies can PRP’s role in equine OA be fully understood and optimized for clinical use.

Additional limitations include the variability in outcome definitions and assessment methods across studies, which complicates direct comparisons. Most trials had short follow-up periods, generally less than 12 months, limiting the evaluation of long-term treatment effects. The predominance of studies reporting positive results raises the possibility of publication bias, potentially overestimating the benefits of platelet-rich plasma therapy. Moreover, heterogeneity in horse populations regarding age, discipline, and management conditions may restrict the generalizability of the conclusions. Finally, the lack of comparable quantitative data due to methodological heterogeneity precluded the performance of a meta-analysis.

Another important limitation of current evidence is the lack of economic evaluations comparing PRP to conventional intra-articular therapies such as corticosteroids or hyaluronic acid. Considering the cost of regenerative products, future studies should also address cost-effectiveness to support clinical decision-making.

## 5. Conclusions

Overall, intra-articular PRP appears to be a safe and potentially effective therapy for equine OA, with only transient and self-limiting inflammatory responses reported. However, evidence on its long-term efficacy and disease-modifying potential remains limited. Future large-scale trials with standardized protocols are needed, ideally with long-term follow-up and cost-effectiveness analyses against conventional therapies.

## Figures and Tables

**Table 1:** PICO Framework for research questions.

Question	Population (P)	Intervention (I)	Comparison (C)	Outcomes (O)	Timeframe
1. Safety	Healthy horses, horses with naturally occurring osteoarthritis (OA), and horses with induced synovitis or OA	Intra-articular injection (IAI) of any platelet-rich plasma (PRP) type (pure P-PRP (P-PRP)/leukocyte and PRP (L-PRP))	Saline solution, L-PRP, and P-PRP	- Adverse events (effusion, lameness) - Synovial fluid biomarkers - Cytological changes	Short term (≤1 month)
2. PRP Types	Healthy horses, horses with naturally occurring OA, and horses with induced synovitis or OA	L-PRP vs. P-PRP preparations	L-PRP vs. P-PRP	- Lameness scores - Synovial effusion - Return to work rates	Short/long term (1–12 months)
3. Composition	Healthy horses, horses with naturally occurring OA, and horses with induced synovitis, or OA	PRP with varying platelet or leukocyte concentrations	L-PRP vs. P-PRP	- Clinical improvement correlated with cellular content - Growth factor and cytokine concentrations	Medium term (1–6 months)
4. Activation	Healthy horses, horses with naturally occurring OA, and horses with induced synovitis, or OA	Activated PRP (CaCl_2_/thrombin) vs. non-activated PRP	Saline	- Inflammatory markers—Clinical safety endpoints	Short term (≤48 h post-IAI)
5. Dosing	Healthy horses, horses with naturally occurring OA, and horses with induced synovitis, or OA	Variable volumes and administration frequencies (single vs. multiple injections)	Standardized volumes (e.g., 5 mL)	- Lameness resolution - Synovial fluid parameters - Owner-reported outcomes	Long term (≥1 year)
6. Efficacy	Healthy horses, horses with naturally occurring OA, and horses with induced synovitis, or OA	PRP monotherapy	Conventional therapies (NSAIDs, corticosteroids)	- Lameness grade (AAEP scale) - Radiographic progression - Athletic performance	Short/medium/long term

**Table 2:** Specific aspects addressed in the inclusion criteria.

Aspect	Description
Safety of intra-articular PRP injections	Studies assessing the safety profile, including any adverse events
PRP type	Studies comparing or reporting the use of P-PRP versus L-PRP, evaluating their effectiveness in clinical or experimental settings
Platelet and leukocyte concentrations	Studies investigating optimal cellular concentrations, including details of preparation methods
PRP activation	Studies examining whether PRP was activated prior to injection, the methods used, and their impact on efficacy
Dosing regimen	Studies reporting the volume, frequency, and administration details of PRP treatment
Clinical outcomes	Studies reporting short-term (1–3 months), medium-term (3.1–12 months), and long-term (≥12 months) outcomes following PRP administration

Acronyms like in Table 1.

**Table 3:** Case series evaluating different PRP formulations in equine naturally occurring OA.

Authors and Date	Study Type, Number of Animals, and Objectives	Study Design and Type of PRP Evaluated	Results and Adverse Effects	Observations	Overall Outcome
Carmona et al. 2007 [36]	Case series. 4 horses with OA. The aim was to evaluate the degree of lameness, joint effusion, and some synovial fluid parameters.	Four horses, including 1 with fetlock OA, 1 with coffin joint OA, 1 with tibiotarsal and intertarsal joint OA, and 1 with femorotibial joint OA, were treated with 3 intra-articular injections (IAI) of leukocyte and PRP (L-PRP) at 2-week intervals in volumes of 10–25 mL. Clinical outcomes were evaluated one year after completion of treatment.	The most marked improvement was observed 2 months after the last treatment and appeared to be sustained for 8 months. No adverse clinical signs resulted from this treatment.	The study lacks controls, The clinicians were not blinded at the time of treatment.	Positive
Pichereau et al. 2014 [37]	Case series. 20 horses with fetlock OA refractory to corticosteroids and rest. The objective was to evaluate the effect of an autologous PRP on cytological changes and IL-1β concentrations in the synovial fluid and the degree of lameness of the treated horses.	Twenty horses were treated, of which 7 had OA of the right anterior fetlock and 13 had OA of the left anterior fetlock. The cellular and biochemical parameters of the synovial fluid and the degree of lameness were determined during three consecutive injections of 3 mL of pure PRP (P-PRP) with a two-week interval between applications. Clinical results were evaluated one year after the end of treatment.	After IAI of PRP, there was a significant reduction in synovial IL-1β concentrations and of the degree of lameness in the treated horses. After one year, 80% of the horses had resumed their usual level of work or competition.	The study lacks controls, The clinicians were not blinded at the time of treatment.	Positive
Mirza et al. 2016 [38]	Case series. 12 horses with OA of the fetlocks or carpus. The study aimed to evaluate the effect of PRP, obtained by gravitational filtration, on biomechanical parameters related to lameness and radiological degree of OA severity.	The study evaluated the effect of an autologous L-PRP. Kinematic and radiological parameters were evaluated before L-PRP IAI and at 6- and 16 weeks post-treatment. Horses were treated with intraarticular volumes of L-PRP between 5 and 10 mL.	Ten of the 12 horses included in the study responded to local anesthetic IAI. Of these 3 responded to PRP therapy at 6 and 16 weeks. The treatment did not affect the radiological recordings	Non-randomized study with little clarity on the joints treated per horse. Study with possible low statistical power.	Positive
Park et al. 2022 [40]	Case series. The effect of PRP in 9 horses with OA (5 mL/joint) on the degree of lameness was evaluated from 1 to 5.	The study evaluated the effect of 5 mL of an autologous P-PRP in 9 horses with OA. The horses were treated 2 times with a 2-week interval between treatments. The degree of lameness was evaluated before and 1–3 months after treatment.	Horses with OA presented an average lameness grade of 2.1 before treatment and subsequently presented a significant improvement by achieving an average lameness grade of 1.1. Follow-up was conducted over 2–3 months.	The affected joints are not described. Data analysis was performed by the authors of the systematic review.	Positive

**Table 4:** Clinical cases evaluating different PRP formulations in equine naturally occurring OA.

Authors and Date	Study Type, Number of Animals, and Objectives	Study Design and Type of PRP Evaluated	Results and Adverse Effects	Observations	Overall Outcome
Bertone et al. [44]	Controlled randomized clinical trial. 40 horses with naturally occurring OA divided in two groups, control and experimental. The aims of the study were to evaluate the effect of the IAI of PRP on subjective lameness score, kinetic gait analysis, radiography and questionary to the owners.	20 horses received intra-articularly 5–6 mL of PRP and 20 horses received 5 mL of saline solution. The horses were evaluated by subjective lameness scoring (0 to 5) at days 0, 7, and 14, kinetic gait analysis (days −1, 7, and 14), joint signs of pain and swelling assessments (days 0, 4, 7, 10, and 14), joint fluid and blood analysis (days 0 and 14), and radiography (days −1 and 14). Horses were exercised twice weekly on a treadmill (at days −1, 4, 7, 10, and 13). The horses were evaluated by the clients via a questionnaire for lameness, comfort, and adverse events before and 12 and 52 weeks after the PRP treatment.	The PRP group had significant improvements in lameness grade, asymmetry indices of vertical peak force, and range of joint motion by 14 days, compared with baseline or control group values. Clients assessed lameness and comfort as improved at 12 and 52 weeks. The APS had greater likelihood (OR, 4.3 to 30.0) of a therapeutic response in horses with a lameness score < 4, <10% vertical force asymmetry, or absence of marked osteophyte formation, subchondral sclerosis, or joint space narrowing. No adverse effects associated with PRP treatment were evident.	Study with unbalanced distribution of affected joints and limbs.	Positive
Smit et al. [39]	Clinical trial. 10 horses, of which 5 had naturally occurring OA of the carpus or fetlocks and 5 were clinically healthy. The study aimed to evaluate the effect of the IAI of PRP on the degree of lameness and synovial effusion, cytological changes, and the concentration of PDGF-BB and TGF-β_1_ in the synovial fluid of the treated horses. In addition, the systemic effect of the treatment was evaluated by periodic hemograms and measurement of total protein and serum amyloid A (SAA).	The study evaluated the effect of a one single IAI of autologous L-PRP (4 mL). The degree of lameness and synovial effusion, the performance of hemograms, the determination of total proteins and SAA, as well as the measurement of cytological changes in synovial fluid of the treated horses were performed before starting the treatment and at days 1, 2, 5, 21 and 56 post-treatment. Synovial mediators were only measured on days 1 and 5.	Horses with OA presented greater synovial effusion than healthy horses before starting treatment; in the latter, there was a significant increase in synovial effusion during the first two days after PRP injection. Both groups presented increased leukocyte concentrations during the first two days in synovial fluid after PRP injection. Synovial concentrations of PDGF-BB and TGF-β_1_ at day 1 significantly increased in the group of healthy horses concerning those with OA. However, on day 5 the same difference was not observed. No differences were observed for SAA values in the study horses.	Study with little clarity about the joints affected in the group of horses with OA. Investigators were not blinded to treatment. No medium or long-term results were described.	Positive

**Table 5:** Experimental studies evaluating different PRP formulations on different healthy equine joints.

Authors and Date	Study Type, Number of Animals, and Objectives	Study Design and Type of PRP Evaluated	Results and Adverse Effects	Observations	Overall Outcome
Textor et al. [41]	Experimental Study (ES) with 7 healthy animals. The study aimed to evaluate the effect of an IAI of three types of PRP and saline solution on synovial fluid concentrations of PDGF-BB, TGF-β_1_, IL-1β, IL-6, and TNF-α.	The 4 fetlocks of each horse were randomly treated with L-PRP. The treatment groups were resting L-PRP (RL-PRP), Ca-activated L-PRP (CL-PRP), and thrombin-activated L-PRP (TL-PRP). Synovial mediator values were taken at 0, 6, 24, 24, 48 and 96 h.	After a single IAI with the different types of L-PRP (2.5 mL/articulation), synovial concentrations of TGF-β_1_ increased significantly compared to baseline concentrations. Synovial concentrations of TNF-α and IL-6 increased significantly after TL-PRP injection, indicating synovial inflammation.	Randomized joint assignment, investigator blinded at the time treatments were performed. Control with saline solution	Neutral
Textor & Tablin, [42]	ES with 7 healthy animals. The objective was to evaluate the effect of an IAI of three types of PRP and saline solution on the clinical response of the joints, as well as changes in the synovial fluid and hemogram characteristics of the treated horses	The 4 fetlocks were randomly treated with autologous L-PRP. The treatment groups were RL-PRP, CL-PRP, TL-PRP. Clinical assessments, hemogram, and synovial fluid values were taken at 0, 6, 24, 48, and 96 h.	After a single IAI with the different types of L-PRP (2.5 mL/joint), it could be observed that TL-PRP produced a greater synovial effusion and a significant increase in leukocyte counts in the synovial fluid of the treated fetlocks compared to the other types of PRP and saline	Randomized joint assignment, investigator blinded at the time treatments were performed. Control with saline solution.	Neutral
Moraes et al. [43]	ES with 8 healthy horses. The study aimed to evaluate the effect of the IAI of PRP on cytological changes and synovial fluid concentrations of TNF-α, IL-1β, IL-1ra, PGE_2_, hyaluronic acid (HA), and chondroitin sulfate (CS) and degrees of synovial effusion and lameness.	The study was divided into 2 phases, of which one was early (EP) and the second prolonged (PP). In the EP a randomly selected fetlock from each horse was treated with 4 mL of autologous L-PRP, and the contralateral joint was treated with physiological saline solution. Synovial fluid samples were collected before treatment and at 3, 6, 24, 48, and 168 h after treatment. At PP one fetlock from each horse was randomly treated with PRP and the synovial fluid samples were collected before starting treatment and at days 7, 14, 21, and 28 post-treatment.	During EP it was observed that IAI of 4 mL of autologous L-PRP, without activation, produced a significant increase in the concentration of leukocytes, total protein, and PGE_2_ than fetlocks treated with saline solution without comparatively affecting the concentration of TNF-α, IL-1β, IL-1ra, HA, and CS. In PP, no treatment significantly affected the parameters evaluated, including clinical variables.	Randomization of joints. Control with saline solution.	Neutral
Usimaki et al. [46]	ES with 18 healthy horses. The objective was to investigate the effects of a single IAI of PRP in horses with interleukin-1β (IL-1β)-induced synovitis by clinical assessment (joint effusion and subjective lameness examination), objective lameness measuring, cytology of synovial fluid, and determination of IL-1β, IL-6, IL-10, IL-17a, interferon (IFN)-γ, TNF-α, PGE2 in synovial fluid and SAA. In addition, Gross pathology and synovial membrane histopathology scoring was performed on PRP-treated, untreated control and normal tarsocrural joints.	12 horses received intra-articularly 3 mL of PRP and 6 horses received 3 mL SS. Synovitis was unilaterally induced in a tarsocrural joint of each horse using recombinant equine IL-1β. Joint effusion, subjective and objective lameness examination, synovial fluid cytology, mediator measurement in synovial fluid were performed at days 0, 1 (induction of synovitis) 2, 4, 7, and 14. SAA was measured at days 2 and 14.	PRP did not decrease lameness or joint circumference compared with untreated controls. Synovial fluid parameters were not different between treatment groups. PRP treatment did significantly decrease gross and histopathology scores.	Main limitations included the use of an induced model of the synovitis, inter-horse variability in the response to IL-1β and likely variability in the constituents of APS from individual horses. Unbalanced design.	Neutral
Page et al. [45]	ES with 5 healthy horses. The aim was to compare metabolic effects between intra-articular triamcinolone acetonide (TA) and PRP by measuring ACTH, cortisol, glucose, insulin, and thyroid hormone analysis, in addition to thyrotropin-releasing hormone (TRH) and oral sugar tests (OSTs) at hours −24, 0, 2, 4, 8, 12, 24, 32, 48, 72, 96, 120, 144, 168, and 336 h	The study used five metabolically normal geldings in a three-way crossover design, comparing intra-articular (metacarpophalangeal joint) saline (3 mL), PRP (3 mL), and 9 mg triamcinolone acetonide (TA). Treatments were randomly assigned, and metabolic parameters (ACTH, cortisol, glucose, insulin, thyroid hormones) were measured via dynamic (TRH, OST) and resting tests over 28-day blocks.	The study found that intra-articular TA significantly suppressed ACTH and cortisol (2–96 h), increased glucose (12–48 h), and caused hyperinsulinemia (peaking at 32 h). It also altered TRH and OST results at 48 h. PRP showed no metabolic effects.	This study did not report any musculoskeletal effect of PRP or information about synovial fluid parameters. However, suggests that the intra-articular treatment with PRP may be safer for horses, particularly those with metabolic conditions like insulin dysregulation or Cushing’s syndrome	Positive

**Table 6:** Quality characteristics of platelet-rich plasma products used in the case series and clinical studies.

	Characteristic (C)									
Author	C1	C2	C3	C4	C5	C6	C7	C8	C9	C10
Carmona et al. [36]	AUT	3.2% SC, 25 mL of fresh blood to produce 2.5 mL of PRP.	Double centrifugation	1st C: 120 g/5 min 2nd C: 240 g/5 min	PRP collected from buffy coat. L-PRP	NR	PLT: 250 ± 71.8 × 10^3^/mL WBC: 8.68 ± 3.78 × 10^3^/mL. TGF-b_1_: 12.5 ± 2.4 ng/mL. Flow cytometry	NR	Prior PRP activation with CaCl_2_	IAI, 3 times each 2 weeks. 10–25 mL of PRP
Pichereau et al. [37]	AUT	SC (0.129 mol/L), 80 mL of fresh blood	Triple centrifugation	1st C: 120 g/5 min 2nd C: 260 g/5 min 3rd C: 1000/10 min	PRP collected from buffy coat. P-PRP	NR	PLT: 560 ± 62 × 10^3^/mL WBC: Scarce. PDGF-BB: 1280 ± 70.91 pg/mL. Impedance count and flow cytometry.	NR	Non activated	IAI, 3 times each 2 weeks. 3 mL of PRP
Bertone et al. [44]	AUT	ACD, 55 mL of fresh blood to produce 5.5 mL of PRP.	Double centrifugation	1st C: 15 min 2nd C: 2 min mixed with polyacrylamide beads	NStride Arthritis Treatment, Biomet Biologics, IN, USA. L-PRP	PLT: 151 ± 13 × 10^3^/mL WBC: 6.2 ± 0.3 × 10^3^/mL IL-1ra: 303 ± 175 pg/mL STNFr1: 4.6 ± 0.1 pg/mL IL-10: 970 ± 479 ng/mL	PLT: 243 ± 53 × 10^3^/mL WBC: 75 ± 4.8 × 10^3^/mL IL-1ra: 1757 ± 100 pg/mL STNFr1: 16.9 ± 2.1 pg/mL IL-10: 3271 ± 807 ng/mL.	1.6 X	Non activated	IAI, only 1 dose of 5–6 mL of PRP
Mirza et al. [38]	AUT	ACD-A (5 mL), 55 mL of fresh blood	Gravitational filter system	Filtration over 10 min	E-PET set. Pall Corporation filter, NY, USA. L-PRP	NR	PLT: 658 ± 219 × 10^3^/mL WBC: 10.66 ± 4.18 × 10^3^/mL. No growth factors were measured	5.2 X	Non activated	IAI, only 1 dose. 5–10 mL of PRP
Smit et al. [39]	AUT	ACD-A (5 mL), 55 mL of fresh blood	Gravitational filter system	NR	E-PET set. Pall Corporation filter, NY, USA. L-PRP	PLT: 130 ± 26 × 10^3^/mL WBC: 8.9 ± 2.0 × 10^3^/mL. No growth factors were measured. Flow cytometry	PLT: 621 ± 200 × 10^3^/mL WBC: 18.7 ± 4.5 × 10^3^/mL. No growth factors were measured. Flow cytometry	4.7	Non activated	IAI, only 1 dose of 4 mL of PRP
Park et al. [40]	AUT	NR	Single centrifugation Semi-automated kit	1500 g/5 min	Arthrex ACP system, FL, USA, P-PRP	PLT: 178 × 10^3^/mL WBC: 6.43 × 10^3^/mL. Flow cytometry	PLT: 448 × 10^3^/mL RBC: 0.25 × 10^3^/mL WBC: Scarce. Flow cytometry	2.5 X	Non activated	IAI, 2 times each 2 weeks of 5 mL of PRP

C1: The source of blood whether autologous (AUT) or allogeneic (ALL). C2: The anti-coagulant, volume and age of the blood used to prepare PRP. C3: The method used to prepare the PRP. C4: The centrifugation conditions (g value, temperature, and time) used in the laboratory or in commercial PRP preparation devices. C5: A complete description of how the PRP was harvested (i.e., from buffy coats or PRP supernatants) and, if a commercial preparation device was used to include its commercial brand. C6: A measurement of the cellular content of the original whole blood including platelet count, white blood cell count, and red blood cell count. C7: A measure of the quality of the PRP preparation (i.e., cell content, platelet activation status, platelet-specific proteins and growth factor content. C8: The concentration factor and yield of platelets obtained. C9: Whether the PRP was activated prior to use, including the substance used to activate the platelets, and C10: The method and number of in vivo applications, the specific sites of application and the volume of PRP administered. ACD-A, acid citrate dextrose solution A. C, centrifugation; IAI, intra-articular injection; NA: not applicable; NR, no reported by the authors. PLT, platelet; WBC: white blood cell.

**Table 7:** Quality characteristics of platelet-rich plasma products used in the controlled experimental studies.

	Characteristic (C)									
Author	C1	C2	C3	C4	C5	C6	C7	C8	C9	C10
Textor et al. [41]	AUT	NR	Gravitational filter system	NR	E-PET set. Pall Corporation filter, NY, USA. L-PRP	NR	PLT: 650 ± 246 × 10^3^/μL WBC: 14.8 ± 3.84 × 10^3^/μL. PDGF-BB: R-PRP 470 ± 870 pg/mL, C-PRP: 2271 ± 1754 pg/mL and TL-PRP: 3811 ± 2942 pg/mL. TGF-β_1_: RL-PRP: 954 ± 1802 pg/mL, CL-PRP: 1577 ± 939 pg/mL and TL-PRP: 3830 ± 1910 pg/mL.	NR	Bovine thrombin (1 U/mL), CaCL _2_ (23 mM)	IAI, only 1 dose of 2.5 mL of PRP
Textor & Tablin. [42]	AUT	ACD-A (5 mL), 55 mL of fresh blood	Gravitational filter system	NR	E-PET set. Pall Corporation filter, NY, USA. L-PRP	PLT: 166 ± 30.9 × 10^3^/μL WBC: 6.87 ± 1.75 × 10^3^/μL.	PLT: 542 ± 196 × 10^3^/μL WBC: 13.1 ± 3.46 × 10^3^/μL. PDGF-BB y TGF-β_1_: data similar to the previous publication for the 3 types of PRP evaluated.	3.2 X	Bovine thrombin (1 U/mL), CaCL_2_ (23 mM)	IAI, only 1 dose of 2.5 mL of PRP
Moraes et al. [43]	AUT	SC, 24 °C	Double centrifugation	1st C: 150 g/5 min 2nd C: 800 g/5 min	NA. L-PRP	NR	PLT: 423 × 10^3^/μL WBC: 8.36 × 10^3^/μL IL-1ra (pg/mL) 57.5 ± 18.1	NR	Non-activated	IAI, only 1 dose of 4 mL of PRP
Usimaki et al. [46]	AUT	ACD, 55 mL of fresh blood to produce 3 mL of PRP	Double centrifugation	1st C: 3200 rpm/15 min 2nd C: 2000 rpm/2 min mixed with polyacrylamide beads	APS; Pro-Stride^®^, Zoetis, NJ USA. L-PRP	NR	NR	NR	NR	IAI, only 1 dose of 3 mL of PRP
Page et al. [45]	AUT	60 mL of fresh blood to produce 3 mL of PRP	NR	NR	APS; Pro-Stride^®^, Zoetis, NJ USA. L-PRP	NR	NR	NR	NR	IAI, only 1 dose of 3 mL of PRP

Acronyms as in Table 3.

**Table 8:** Qualitative scoring of the PRP used across the studies.

			Characteristic (C)										Overall Score
Study	Type of Study	PRP Type	C1	C2	C3	C4	C5	C6	C7	C8	C9	C10	
Carmona et al. [36]	Case series	L-PRP	10	10	10	10	10	0.0	10	0.0	10	10	80
Pichereau et al. [37]	Case series	P-PRP	10	10	10	10	10	0.0	10	0.0	10	10	80
Mirza et al. [38]	Case series	L-PRP	10	10	10	10	10	0.0	10	10	10	10	90
Park et al. [40]	Case series	P-PRP	10	0.0	10	10	10	10	10	10	10	10	90
Smit et al. [39]	RCT	L-PRP	10	10	0.0	10	10	10	10	10	10	10	90
Bertone et al. [44]	RCT	L-PRP	10	10	10	10	10	10	10	10	10	10	100
Textor et al. [41]	CES	L-PRP	10	0.0	10	0.0	10	0.0	10	0.0	10	10	60
Textor & Tablin, [42]	CES	L-PRP	10	10	10	0.0	10	10	10	10	10	10	90
Moraes et al. [43]	CES	L-PRP	10	5.0	10	10	10	0.0	10	0.0	10	10	75
Usimaki et al. [46]	CES	L-PRP	10	10	10	10	0.0	0.0	0.0	0.0	0.0	10	50
Page et al. [45]	CES	L-PRP	10	5.0	0.0	0.0	10	0.0	0.0	0.0	0.0	10	35

RCT, randomized clinical trial; CES, controlled experimental study. Other acronyms as in Tables.

## Data Availability

No new data were created or analyzed in this study. Data sharing is not applicable to this article.

## Abbreviations

The following abbreviations are used in this manuscript:

AAEP	American Association of Equine Practitioners
ACD/ACD-A	Acid Citrate Dextrose A
ACTH	Adrenocorticotropic Hormone
ALL	Allogeneic
APS	Autologous Protein Solution
AUT	Autologous
CaCl_2_	Calcium Chloride
CES(s)	Controlled Experimental Studies
CL-PRP	Calcium-activated Leukocyte-rich Platelet-Rich Plasma
CS(s)	Case Series
GFs	Growth Factors
HA	Hyaluronic Acid
IAI	Intra-Articular Injection
IFN-γ	Interferon gamma
IL-1β	Interleukin 1 beta
IL-6	Interleukin 6
IL-10	Interleukin 10
IL-17A	Interleukin 17A
L-PRP	Leukocyte-rich Platelet-Rich Plasma
NSAIDs	Non-Steroidal Anti-Inflammatory Drugs
OA	Osteoarthritis
OST	Oral Sugar Test
P-PRP	Pure Platelet-Rich Plasma
PDGF-BB	Platelet-Derived Growth Factor-BB
PGE_2_	Prostaglandin E_2_
PICO	Population, Intervention, Comparison, Outcome
PLT	Platelet
PRG	Platelet-Rich Gel
PRISMA	Preferred Reporting Items for Systematic Reviews and Meta-Analyses
PRP	Platelet-Rich Plasma
RBC	Red Blood Cells
RCT(s)	Randomized Clinical Trial(s)
Robvis	Risk-of-bias VISualization
ROBINS-I	Risk Of Bias In Non-randomized Studies of Interventions
RoB 2.0	Revised Cochrane risk-of-bias tool for randomized trials
SAA	Serum Amyloid A
SA	Septic Arthritis
SC	Sodium Citrate
WOS	Web of Science
STNFr1	Soluble Tumor Necrosis Factor Receptor 1
TA	Triamcinolone Acetonide
TGF-β_1_	Transforming Growth Factor beta 1
TNF-α	Tumor Necrosis Factor alpha
TRH	Thyrotropin-Releasing Hormone
WBC	White Blood Cells
